# *Ethiopian Crop Type 2020 (EthCT2020)* dataset: Crop type data for environmental and agricultural remote sensing applications in complex Ethiopian smallholder wheat-based farming systems (Meher season 2020/21)

**DOI:** 10.1016/j.dib.2024.110427

**Published:** 2024-04-14

**Authors:** Gerald Blasch, Yoseph Alemayehu, Louise Lesne, Jolan Wolter, Matthieu Taymans, Tsegaab Tesfaye, Tamirat Negash, Mequanint Andulalem, Kitessa Gutu, Megersa Debela, Zerihun Eshetu, Kindie Tesfaye, Khondoker Mottaleb, Pierre Defourny, David P. Hodson

**Affiliations:** a International Maize and Wheat Improvement Center (CIMMYT), Addis Ababa, Ethiopia; b Earth and Life Institute, Université catholique de Louvain, Louvain-la-Neuve, Belgium; c Ambo Agricultural Research Center, Ethiopia; d Kulumsa Agricultural Research Center, Ethiopia; e Adet Agricultural Research Center, Ethiopia; f Bako Research Center, Ethiopia; g Sinana Agricultural Research Center, Ethiopia; h International Maize and Wheat Improvement Center (CIMMYT), Texcoco, Mexico

**Keywords:** *In-situ* crop type observation, Field reference data, Remote sensing, Crop type mapping, Machine learning, Agriculture, Annual cropland, Ethiopia

## Abstract

Crop type observation is crucial for various environmental and agricultural remote sensing applications including land use and land cover mapping, crop growth monitoring, crop modelling, yield forecasting, disease surveillance, and climate modelling. Quality-controlled georeferenced crop type information is essential for calibrating and validating machine learning algorithms. However, publicly available field data is scarce, particularly in the highly dynamic smallholder farming systems of sub-Saharan Africa. For the 2020/21 main cropping season (*Meher*), the *Ethiopian Crop Type 2020* (*EthCT2020*) dataset compiled from multiple sources provides 2,793 harmonized, quality-controlled, and georeferenced *in-situ* samples on annual crop types (7 crop groups; 22 crop classes) at smallholder field level across the complex and highly fragmented agricultural landscape of Ethiopia. The focus was on rainfed, wheat-based farming systems. A nationwide ground data collection campaign (GDCC; *Source 1*) was designed using a stratification approach based on wheat crop calendar information, and 1,263 *in-situ* data samples were collected in selected sampling regions. This *in-situ* data pool was enriched with 1,530 wheat samples extracted from a) the Wheat Rust Toolbox (WRTB; *Source 2*; 734 samples), a database for wheat disease surveillance data [1] and b) an inhouse farm household survey database (FHSD; *Source 3*; 796 samples). Obtained field data was labelled according to the Joint Experiment for Crop Assessment and Monitoring (JECAM) guidelines for cropland and crop type definition and field data collection [2] and the FAO Indicative Crop Classification [3]. The *EthCT2020* dataset underwent extensive processing including data harmonization, mixed pixel assessment through visual interpretation using 5 m Planet satellite image composites, and quality-control using Sentinel-2 NDVI homogeneity analysis. The *EthCT2020* dataset is unique in terms of crop diversity, pixel purity, and spatial accuracy while targeting a countrywide distribution. It is representative of Ethiopia's complex and highly fragmented agricultural landscape and can be useful for developing new machine learning algorithms for land use land cover mapping, crop type mapping, agricultural monitoring, and yield forecasting in smallholder cropping systems. The dataset can also serve as a baseline input parameter for crop models, climate models, and crop disease and pest forecasting systems.

## Graphical abstract

**Figure:**
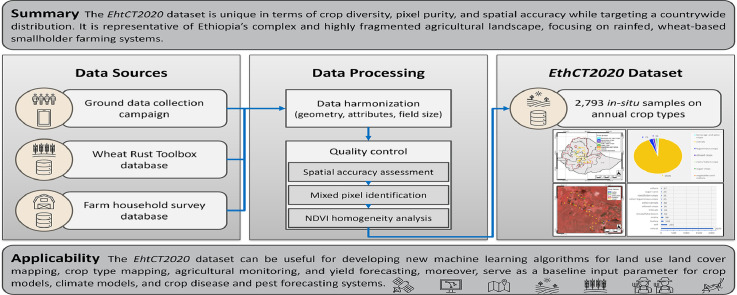

Specifications Table

**Table:** 

Subject	Agriculture Sciences, Earth Science, Computer Science
Specific subject area	Ground Truth, Remote Sensing, GIS, Machine Learning, Crop Type
Type of data	Vector (ESRI shapefile)
Data collection	*In-situ* data collection compiled in a harmonized dataset from multiple sources: (a) CIMMYT-led nationwide ground data collection campaign using ODK and GPS (GDCC; *Source 1*), (b) Wheat Rust Toolbox, a database for field disease surveillance and monitoring using ODK [1] (WRTB; *Source 2*), and (c) CIMMYT farm household survey database (FHSD; *Source 3*). Subsequently, quality control and harmonization using the statistical software R (version 4.1.3) [4] and the GIS software QGIS (version 3.14.16-Pi) [5].
Data source location	Addis Ababa, Amhara, Beneshangul Gumu, Oromia, SNNPR; Ethiopia Geospatial information is in the dataset
Data accessibility	Repository name: Mendeley Data Data identification number: 10.17632/mfpvmk8cnm.1 Direct URL to data: https://data.mendeley.com/datasets/mfpvmk8cnm/1

## 1 Value of the Data

• The first open-access *in-situ* dataset of high-quality crop type information in terms of crop variation, pixel purity, and spatial accuracy at the national scale for Ethiopia, second most populated country in Africa, representative for its complex and highly fragmented smallholder rainfed crop production agricultural landscape, which is useful as field reference data for remote sensing and crop modelling applications.
• This dataset is beneficial for researchers working in environmental and agricultural monitoring and modelling that need field reference (ground truth) data for training and validating machine learning models for classification and mapping.
• This dataset can be useful for the improvement or development of new remote sensing machine learning methods for key environmental and agricultural products such as land use / land cover maps, cropland masks, crop type maps, and vegetation status maps at both regional and national scales, and as a benchmark for comparison of different classification methods.

## 2 Background

Crop disease surveillance and early warning require detailed host (crop) information to have maximum utility. Satellite-based remote sensing can provide the desired host information layers at regional and national scales, i.e., crop type distribution maps. However, mapping crop types in Ethiopia at the national scale remains challenging due to a highly fragmented agricultural landscape and very frequent cloud coverage during the rain-fed main growing season. As a result, high quality crop type maps are not available. The *EthCT2020* dataset was created to address the lack of host information for the Ethiopian Wheat Rust Early Warning and Advisory System. The dataset was used to compute static and dynamic wheat maps at the national scale.

## 3 Data Description

This paper reports the *EthCT2020* dataset, consisting of a vector file in ESRI shapefile format (spatial reference system: WGS84 / UTM zone 37N; EPSG: 32637). The ESRI shapefile format is composed of four files: 1) .shp – the main file that stores the feature geometry, 2) .shx – the index file that stores the index of the feature geometry, 3) .dbf – the dBASE table that stores the attribute information, and 4) .prj – the file that stores the coordinate system information. It stores nontopological geometry and attribute information, and can be opened in the majority of GIS software (e.g., QGIS, ArcGIS) and scientific programming software packages (e.g, R, Python).

The shapefile of this dataset contains the delimitation of 2,793 circular plots (10 m radius) located in cultivated fields, and the crop information (crop group and crop class) of the 2020/21 main Meher season (June 2020 to February 2021) for each field plot. The shapefile's attribute table has 15 columns. The 1^st^ column is the identifier (1^st^ column: *id*), a unique identifier for each field plot. The 2^nd^ to 6^th^ columns store metadata such as source-specific identifiers (2^nd^: *id_src1*/*id_source1*; 3^rd^: *id_src2*/*id_source2*; 4^th^: *id_src3*/*id_source3*), specific source name (5^th^: *sorc_nm*/*source_name*), and data collection timestamp (6^th^: *sub_dat*/*sub_date*). To maintain consistency in the context of the global diversity of crop types, the 7^th^–13^th^ columns follow a hierarchical grouping of crops suitable for sharing common aggregation levels as outlined in the JECAM guidelines [2], which is an adaption from the Indicative Crop Classification developed by FAO in the frame of agricultural census [3]. This grouping is composed of four hierachy levels: land cover category (7th: *lnd_cvr*/*land_cover*), crop group (8th: *id_c_gr*/*id_c_group*; 9th: *c_group*), crop class (10th: *id_c_cl*/*id_c_class*; 11th: *c_class*), and crop sub-class (12th: *id_c_sb*/*id_c_subclass*; 13th: *c_sbcls*/*c_subclass*). The geospatial information of the field plot centroid is stored in two additional columns (14th: *lat*; 15th: *long*). Data records per row correspond to one specific field plot, representing its land use and crop type class during the 2020/21 main season according to the corresponding source.

**Fig. 1:**
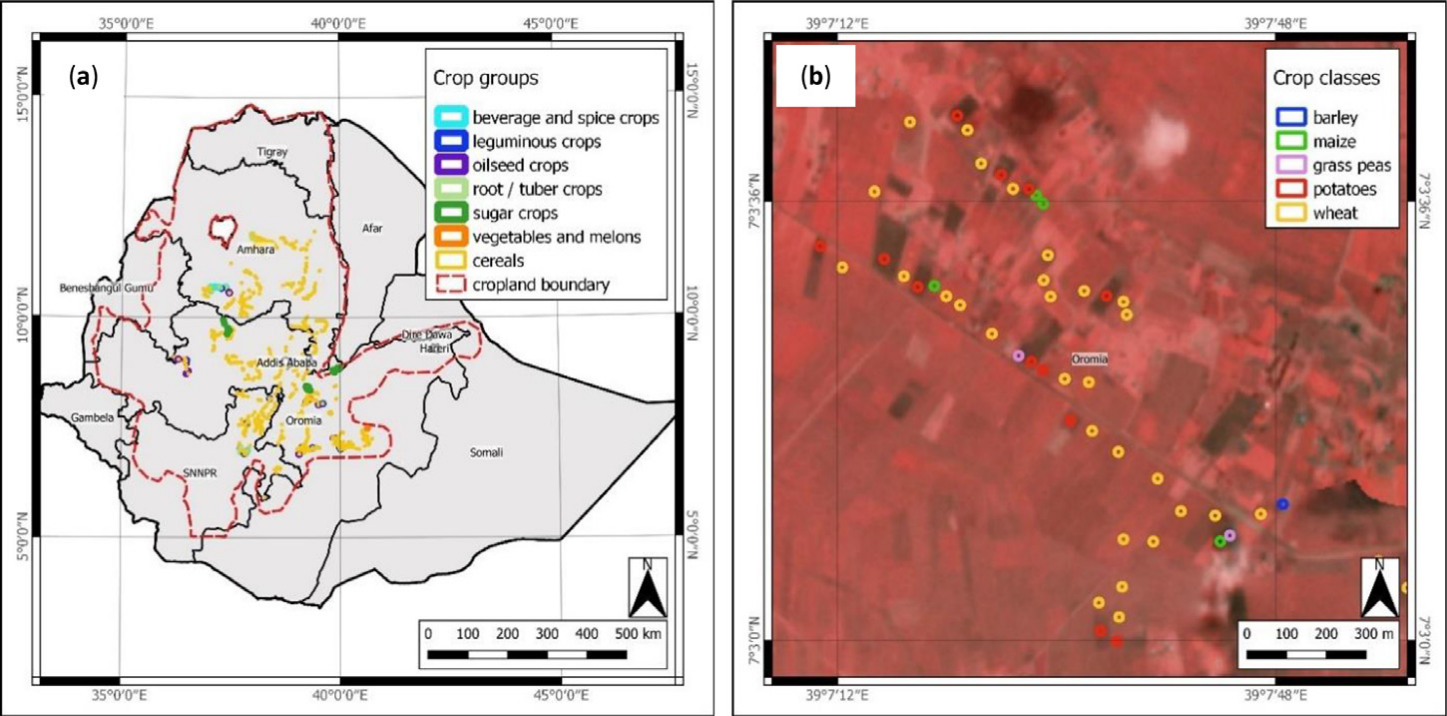
(**a**) Location of the *EthCT2020* dataset and the distribution of the fields, showing crop groups; (**b**) Example of circular field plots showing crop classes over a color-infrared (CIR) composition of PlanetScope Surface Reflectance Mosaics (NIR-red-green bands) from September 2020 at 4.8 m spatial resolution (West Arsi zone, Oromia region).

**Fig. 2:**
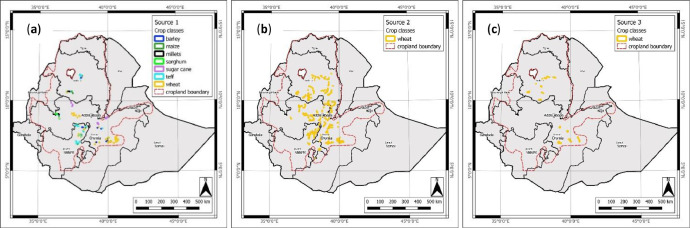
Location of the source datasets and the distribution of the fields, showing crop classes (**a**) *Source 1* – Ground Data Collection Campaign (GDCC); (**b**) *Source 2* – Wheat Rust Toolbox (WRTB); and (**c**) *Source 3* – Farm Household Survey Database (FHSD).

**Fig. 3:**
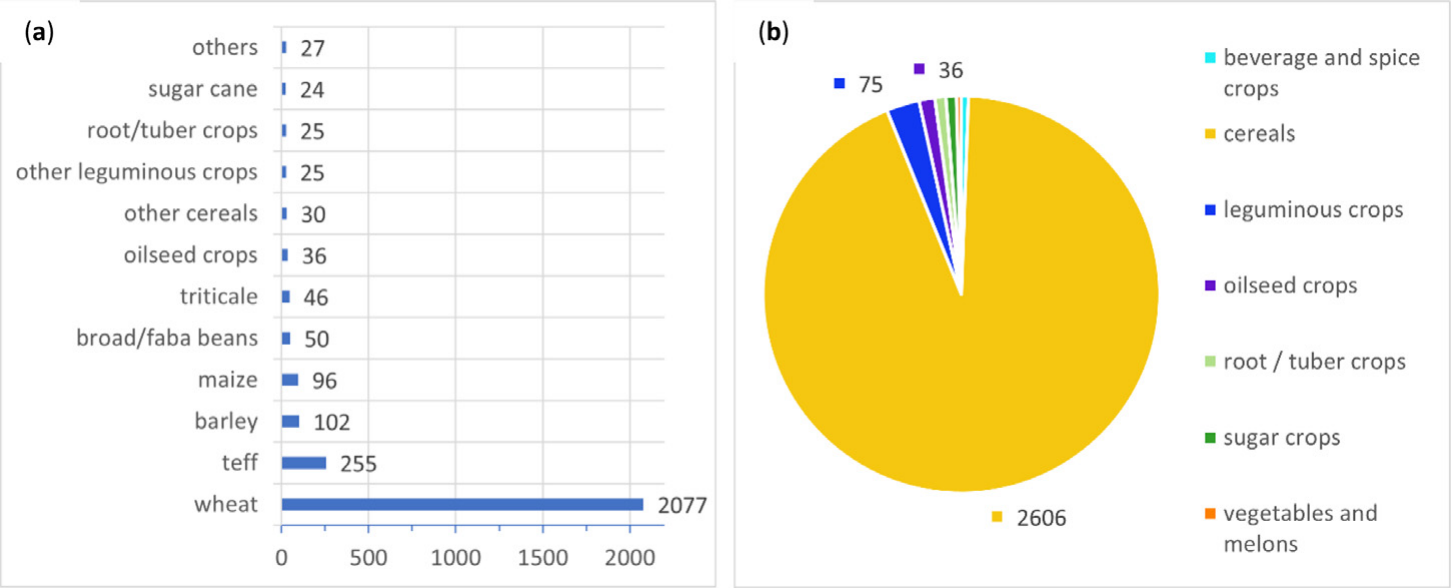
(**a**) Distribution of crop groups (samples per crop group; crop groups with <35 samples are not displayed); (**b**) Distribution of main crop classes (samples per crop class). *Note:* The *EthCT2020* dataset contains 13 circle field plots, having a high overlap (≥10 m) with neighboring circle field plots. The IDs (*id*) are: 579, 583, 585, 1064, 2113, 2119, 2161, 2201, 2287, 2336, 2340, 2350, and 2362. All overlapping polygons belong to the wheat crop class (*c_class*).

Following the JECAM legend for crop types and land cover classes [2], all compiled samples belong to the *annual cropland* land cover class and were assigned into seven crop groups (*cereals; vegetables and melons; oilseed crops; root/tuber crops with high starch or inulin content; beverage and spice crops; leguminous crops*; and *sugar crops*), 22 crop classes (*wheat; maize; teff; sorghum; barley; triticale; oats; millets; leafy or stem vegetables; fruit-bearing vegetables; root, bulb, or tuberous vegetables; groundnuts; other oilseed crops; potatoes; sweet potatoes; spice crops; broad/faba beans; chick peas; lentils; peas; other leguminous crops*; and *sugar cane*), and 8 sub-classes (*cabbages; tomatoes; onions; linseed; Niger seed; chillies and peppers; grass peas*; and *haricot beans*). As teff is a highly important and predominant staple cereal in Ethiopia, teff and triticale were listed as specific cereal crop classes within the cereal group, in contrast to JECAM. Fig. 1, Fig. 2 show the location of the *EthCT2020* dataset and an example of circular field plots showing crop class information in the Oromia region. Distribution of crop groups and classes is shown in Fig. 3.

## 4 Experimental Design, Materials and Methods

### 4.1 Area of data compilation and collection

**Fig. 4:**
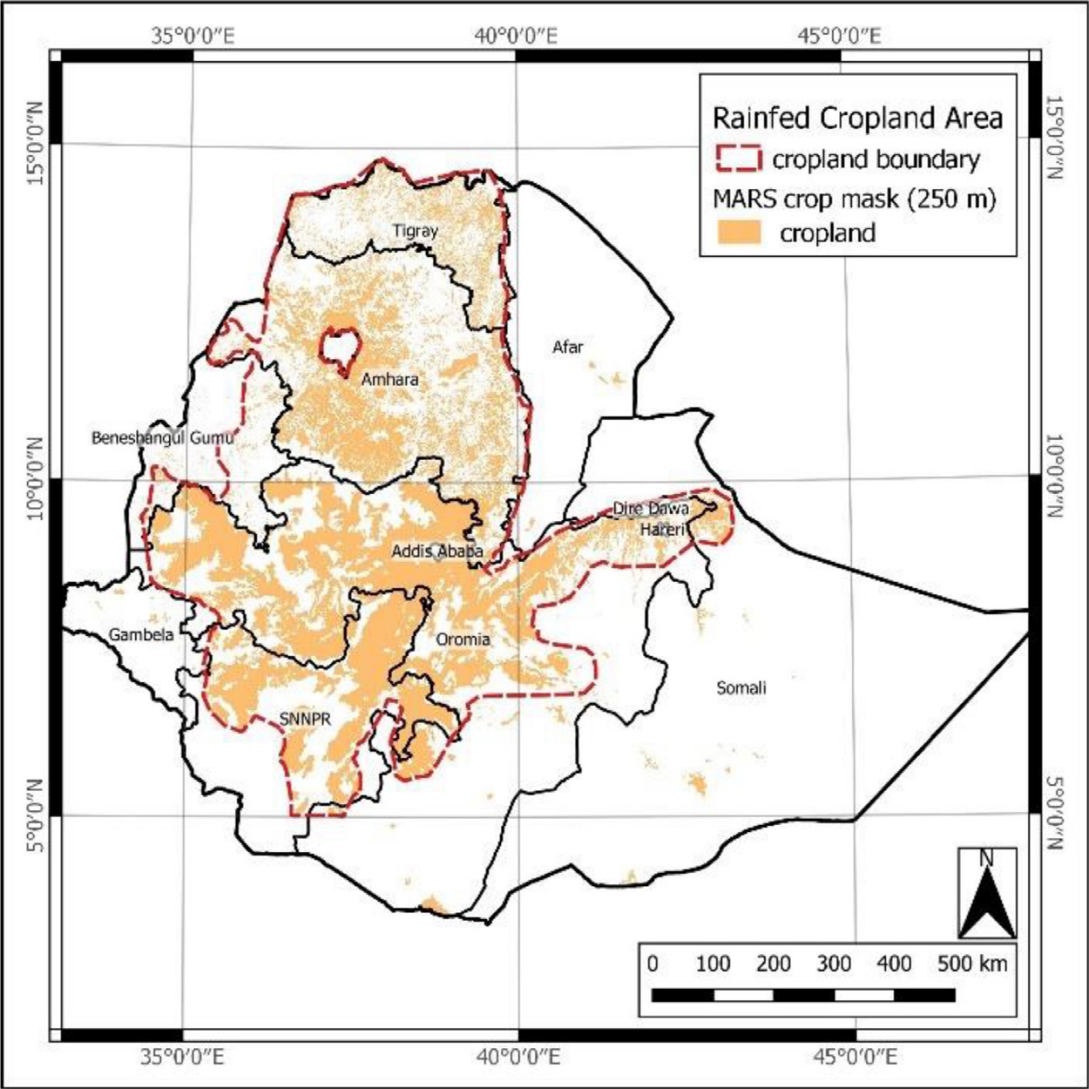
Overview of the rainfed cropland area of Ethiopia – African MARS-JRC crop mask over Ethiopia (orange) overlayed with the vectorized, generalized cropland boundary (red dashed line).

The target area of the *EthCT2020* dataset is the rainfed cropland of Ethiopia (latitudes: 3° 23’–14° 53’ N; longitudes 32° 59’–47° 58’ E). For its delimitation, the boundary of the African MARS-JRC crop mask raster product (spatial resolution of 250 m) [6] was manually generalized into a polygon file in ESRI shapefile format (spatial reference system: WGS84 / UTM zone 37N; EPSG: 32637) (Fig. 4), covering the highland areas, upper rift valley, and to some extent the western humid lowlands. This simplified spatial cropland layer was used to compile the *EthCT2020* dataset as well as to design a ground data collection campaign for creating the *Source 1* dataset.

### 4.2 *Source 1*: Ground Data Collection Campaign (GDCC; 1,263 samples)

**Fig. 5:**
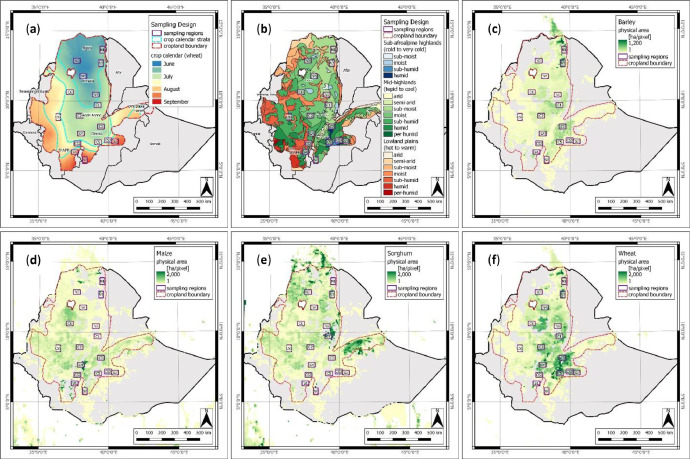
Location of sampling regions in relation to – (**a**) the crop calendar (planting date) sampling frame; (**b**) the agro-ecological zones [8]; and (**c-f**) the physical area of barley, maize, sorghum, and wheat (pixel size: 100 km^2^) [10].

A comprehensive ground data collection campaign was designed as a stratified windshield survey following the JECAM sampling strategy [2]. Due to the seasonality of rainfall patterns, the crop calendar was identified as the most relevant information to create representative and feasible strata. Four strata were delineated from a crop calendar dataset computed from main-season wheat planting dates at district level using geostatistical interpolation (ordinary kriging) and unsupervised (*k* means) classification methods. In total, 17 sampling regions were placed across all strata, considering information on proportion and density of major cereal crops as well as sub-national agricultural statistics [7] and local expert knowledge, to ensure a representative set of crop production-relevant agro-ecological zones [8], rainfall pattern [9], and key production areas of cereal crops (e.g., maize, sorghum, wheat, and barley) according to MapSPAM data [10] (Fig. 5).

For data collection, multiple fieldwork campaigns were conducted by partners from regional and national research centers between September 2020 and February 2021. Circumstances for fieldwork activities were difficult due to Covid-19 and related inter and intraregional travel restrictions, political tensions (in e.g., Oromia, Amhara, and Tigray), and the presence of the locust epidemic in southern Ethiopia. Thus, data could only be collected from 10 sampling regions (N4, C2-6, CS1-3, and S3), encompassing main cereal rainfed production areas. The open-source mobile data collection platform Open Data Kit (ODK) was used to capture geographic coordinates from at least 10 m inside the field, cropping system, and crop type information from fields with a minimum size of 30 m by 30 m along major and secondary roads. Additionally, local agricultural experts assisted during fieldwork. A polygon vector file (ESRI shapefile format; spatial reference system: WGS84 / UTM zone 37N; EPSG: 32637) was created by applying a 10 m buffer around recorded coordinates, resulting in circular field plots of 10 m radius for all surveyed fields (*Source 1* dataset).

### 4.3 *Source 2*: Wheat rust toolbox (WRTB; 734 samples)

Within the Borlaug Global Rust Initiative, the Wheat Rust Toolbox database (hosted by Aarhus University and the Global Rust Reference Center) was developed for global wheat rust surveillance and monitoring to mitigate the global and regional threat of wheat rust diseases to agricultural crop production [1]. The Wheat Rust Toolbox provides up to date information on the status of wheat rust diseases derived from seasonal standardized field surveys. In Ethiopia, wheat rust surveys have been seasonally undertaken, covering approx. 1000 fields per year since 2007. From 2020, expert pathologists record disease relevant data, crop type, and phenology from at least 10 m inside the field using ODK along major and minor roads across wheat production regions.

For enriching the *Source 1* dataset, the crop type and geospatial information of each surveyed field from the 2020/21 main season were extracted from the database [1]. The data was provided by CIMMYT in a comma-separated values (.csv) file containing 53 columns including meta information (e.g., *location id; latitude*; and *longitude*), crop information (e.g., *host genus name*), field management information, and disease-related information. The records of only four columns (*location id; host genus name; latitude*; and *longitude*) were kept, and column names renamed (e.g., *location id* → *id_source2; host genus name* → *c_class*) to match the JECAM legend definition as described in Section 1. This *Source 2* data (.csv file) was converted into a polygon vector file (ESRI shapefile format; spatial reference system: WGS84 / UTM zone 37N; EPSG: 32637) by applying a 10 m buffer around recorded coordinates, resulting in circular field plots of 10 m radius.

### 4.4 *Source 3*: Farm household survey database (FHSD; 796 samples)

For an impact study on the Ethiopian wheat rust early warning and advisory system [11], a CIMMYT inhouse database was built from a farm household survey carried out in major wheat-producing districts in the Amhara and Oromia regions at the end of the 2020 main cropping season [12]. Besides socioeconomic and demographic information, plot-level crop type and field management information as well as geographic coordinates were collected by trained enumerators from approx. 1,000 wheat farmers.

To complement the *EthCT2020* dataset, the crop type (here: only wheat) and geospatial information of each surveyed field were extracted from the inhouse database. The survey data were provided by CIMMYT in a comma-separated values (.csv) file containing eight columns including the *field id* and GPS readings from each measured location. The records of only three columns (*field id, latitude_center*; and *longitude_center*) were kept, and column names renamed (e.g., *field id* → *id_source3; latitude_center* → *lat; longitude_center* → *long*) to match the JECAM legend definition as described in Section 1. Additionally, one column for the crop class (*c_class*) was added to the attribute table, and the *wheat* label assigned to each surveyed field plot. This *Source 3* data (.csv file) was converted into a polygon vector file (ESRI shapefile format; spatial reference system: WGS84 / UTM zone 37N; EPSG: 32637) by applying a 10 m buffer around recorded coordinates, resulting in circular field plots of 10 m radius (*Source 3* dataset).

### 4.5 Data processing

To ensure high data quality in terms of location accuracy and sample purity, the compiled multi-source dataset underwent extensive processing consisting of data harmonization, mixed pixel identification and spatial accuracy assessment through visual image interpretation, and quality-control using NDVI homogeneity analysis.

As previously outlined (Sections 2.2, 2.3, and 2.4), all source datasets had the same data structure in terms of feature geometry and attribute table, geospatial reference system, as well as the same field size and geometry. To finalize data harmonization, all three source datasets were merged into one multi-source *in-situ* dataset, and the attribute table was modified by adding and editing information regarding land cover category, crop group, crop class, and crop sub-class according to the JECAM legend definition.

Mixed pixel effects impacting the sample purity are commonly caused by including pixel(s) covering similar land use / land cover objects such as field boundary vegetation (e.g., trees, tree shadows), crops from a neighboring field or other land use / land cover classes such as water bodies, roads, and buildings (including building shadows). For assessing the spatial accuracy of field plots and eliminating mixed pixel effects, a visual image interpretation of spatially high-resolution (4.8 m per pixel) PlanetScope Visual and Surface Reflectance (Analysis Ready) Mosaics was performed for each data record using image enhancement techniques (e.g., contrast stretching and false color composites). Those monthly satellite composite products were provided by Planet Labs (Planet Labs, San Francisco, CA, United States) through the NICFI (Norway's International Climate and Forest Initiative) Data Program for the entire main cropping season June 2020–February 2021. During this location quality assessment, field plot polygons identified with lower quality due to the presence of mixed pixels or low location accuracy were flagged as poor quality.

Subsequently, an additional satellite-based quality control was executed using a Sentinel-2 NDVI homogeneity analysis on the dataset to evaluate the spectral reflectance variation across each field plot polygon according to the logic that the lower the spectral variability the more homogenous the field plot (respectively, the lower the probability of mixed or non-crop pixels). A Sentinel-2 satellite time-series (743 images) was computed for the 2020/21 main season through the Sen2Agri system [13,14], resulting in Sentinel-2 Level 2A satellite images, covering the target area (respectively, 95 Sentinel-2 tiles; each tile = 100 km x 100 km). Data records with higher NDVI variation were flagged as poor quality.

Poor quality samples (e.g., very small fields, unsure location, mixed pixels presence, and high NDVI range) obtained from both quality assessments were iteratively visually double-checked against spatially high-resolution satellite imagery (e.g., PlanetScope Visual and Surface Reflectance basemaps), and

(a) shifted to a more centric position inside the field, usually towards the field centroid. The most desirable location was determined through visual image interpretation, ensuring that the new position is free of mixed pixel effects, the sample is completely located within the associated field, and has to receive a homogenous crop type coverage from the associated field with high location precision; and free of mixed pixel effects or
(b) removed (if truly poor quality) from the dataset to obtain the final harmonized high quality *EthCT2020* dataset version which guarantees high location accuracy and sample purity.

Data processing and analyses were performed using the statistical software R, version 4.1.3 [4] and the free and open source geographic information system QGIS, version 3.14.16-Pi [5].

## Limitations

None

## Ethical Statement

The authors confirm that, after reading the ethical requirements for publication in Data in Brief, the current work does not involve human subjects, animal experiments, or any data collected from social media platforms.

## CRediT authorship contribution statement

**Gerald Blasch:** Conceptualization, Writing – original draft, Writing – review & editing, Methodology, Investigation, Visualization, Validation, Supervision. **Yoseph Alemayehu:** Methodology, Data curation, Writing – review & editing. **Louise Lesne:** Methodology, Visualization, Validation. **Jolan Wolter:** Methodology, Visualization, Validation. **Matthieu Taymans:** Conceptualization, Methodology, Validation. **Tsegaab Tesfaye:** Data curation, Writing – review & editing. **Tamirat Negash:** Data curation, Writing – review & editing. **Mequanint Andulalem:** Data curation, Writing – review & editing. **Kitessa Gutu:** Data curation, Writing – review & editing. **Megersa Debela:** Data curation, Writing – review & editing. **Zerihun Eshetu:** Data curation, Writing – review & editing. **Kindie Tesfaye:** Methodology, Validation, Writing – review & editing. **Khondoker Mottaleb:** Methodology, Validation, Writing – review & editing. **Pierre Defourny:** Methodology, Conceptualization, Writing – review & editing. **David P. Hodson:** Supervision, Writing – review & editing, Funding acquisition.

## Data Availability

Ethiopian Crop Type 2020 (EthCT2020) dataset (Original data) (Mendeley Data).
